# *Mytilus galloprovincialis* Myticin C: A Chemotactic Molecule with Antiviral Activity and Immunoregulatory Properties

**DOI:** 10.1371/journal.pone.0023140

**Published:** 2011-08-08

**Authors:** Pablo Balseiro, Alberto Falcó, Alejandro Romero, Sonia Dios, Alicia Martínez-López, Antonio Figueras, Amparo Estepa, Beatriz Novoa

**Affiliations:** 1 Instituto de Investigaciones Marinas (IIM), CSIC, Vigo, Spain; 2 Instituto de Biología Molecular y Celular (IBMC), Miguel Hernández University, Elche, Spain; University of São Paulo, Brazil

## Abstract

Previous research has shown that an antimicrobial peptide (AMP) of the myticin class C (Myt C) is the most abundantly expressed gene in cDNA and suppressive subtractive hybridization (SSH) libraries after immune stimulation of mussel *Mytilus galloprovincialis.* However, to date, the expression pattern, the antimicrobial activities and the immunomodulatory properties of the Myt C peptide have not been determined. In contrast, it is known that Myt C mRNA presents an unusual and high level of polymorphism of unidentified biological significance. Therefore, to provide a better understanding of the features of this interesting molecule, we have investigated its function using four different cloned and expressed variants of Myt C cDNA and polyclonal anti-Myt C sera. The *in vivo* results suggest that this AMP, mainly present in hemocytes, could be acting as an immune system modulator molecule because its overexpression was able to alter the expression of mussel immune-related genes (as the antimicrobial peptides Myticin B and Mytilin B, the C1q domain-containing protein MgC1q, and lysozyme). Moreover, the *in vitro* results indicate that Myt C peptides have antimicrobial and chemotactic properties. Their recombinant expression in a fish cell line conferred protection against two different fish viruses (enveloped and non-enveloped). Cell extracts from Myt C expressing fish cells were also able to attract hemocytes. All together, these results suggest that Myt C should be considered not only as an AMP but also as the first chemokine/cytokine-like molecule identified in bivalves and one of the few examples in all of the invertebrates.

## Introduction

Antimicrobial peptides (AMPs) are small, gene-encoded cationic peptides that constitute important innate immune effectors from organisms spanning most of the phylogenetic spectrum [1], [2]. AMPs have a broad range of actions against many microorganisms [3], [4], including viruses [5], and it is not usual to observe the acquisition of resistance to bacterial strains by these molecules [6]. Moreover, constitutive and induced production of AMPs has been reported, with various expression patterns depending on the species, tissue and cell type and infection or inflammation state. These natural antibiotics (41000 AMPs have been estimated in multicellular organisms) exemplify the complexity and heterogeneity of the innate immune responses because they can directly kill microbes and act as modifiers of innate and even adaptive immune responses [7].

In marine invertebrates, which live in environments with an abundance of potentially pathogenic microorganisms, AMPs are the leading elements of the immune response. For instance, in Mediterranean mussels (*Mytilus galloprovincialis*), which in comparison with other bivalves have almost no massive mortality records, several types of AMPs have been described, among them defensins, mytilins and myticins [8], [9], [10].

Three different isoforms (A, B and C) of mussel myticins have been described so far, with isoform C being the most expressed transcript in adults; to our knowledge, it is the only known mussel AMP expressed at larval stages [11], [12]. Strikingly, although antimicrobial specificity of Myt C has not been demonstrated, it presents higher levels of RNA polymorphism than those previously reported for any other mussel AMP [11]. However, to date, little is known about how this variability is generated and what role it plays in the mussel immune response. Likewise, it has not yet been established whether there is any correlation between Myt C RNA polymorphisms and Myt C protein variants. A plausible hypothesis could be that the set of Myt C molecules might constitute a pathogen recognition receptor (PRR) system because sequence diversity is a key feature of a relatively small number of effector molecules involved in self and non-self recognition [12]. Nevertheless, further studies are needed to confirm this hypothesis.

In this work, we analyzed the *in vivo* tissular and cellular expression patterns of Myt C at the mRNA and protein levels. By expressing a recombinant peptide of Myt C, we demonstrated for the first time its potential antiviral and immunoregulatory properties. Altogether, our results suggest that Myt C might play a significant role in the molluscan immune response against pathogens and external aggressions. Moreover, the activity of recombinant Myt C peptides against fish viruses in fish cell lines also suggests that this AMP is active across species; therefore, it might be used to enhance fish defenses in stressful environments and as a model molecule for improving the design of fish antimicrobial drugs.

## Results

### Tissue distribution and subcellular location of Myt C mRNA and protein

**Figure 1:**
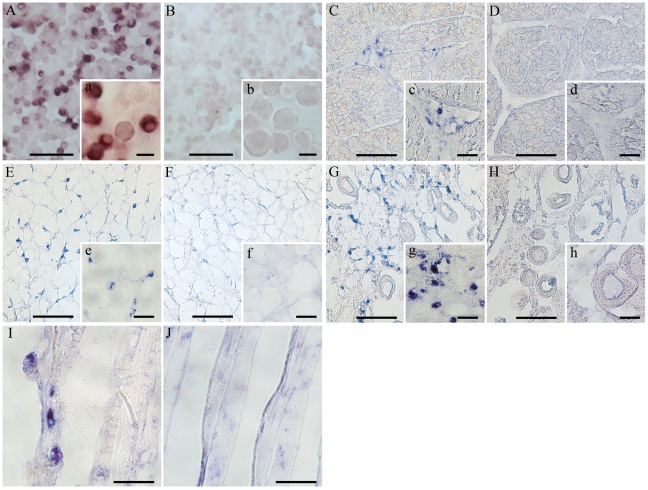
Myt C expression in mussels at the mRNA level. ISH in hemocytes, muscle, connective tissue, gonad and gills from mussel using the myticin antisense RNA probes (A, C, E, G and I, respectively). Control samples were hybridized with the sense probes (B, D, F, H and J, respectively). Scale bar: 50 µm in all cases except in a and b (12.5 µm).

*In situ* hybridization (ISH) assays (Figure 1) showed that hemocytes were the mussel cells with the highest Myt C expression. A hemocyte monolayer hybridized with a Myt C antisense RNA probe is shown in Figure 1A. All of the cells were not marked by the probe, which indicates that some hemocytes do not express Myt C. When this technique was applied to different mussel tissues, such as muscle, connective tissue, gonad and gills (Figure 1C, E, G and I, respectively), the positive reaction was detected mainly in circulating hemocytes.

**Figure 2:**
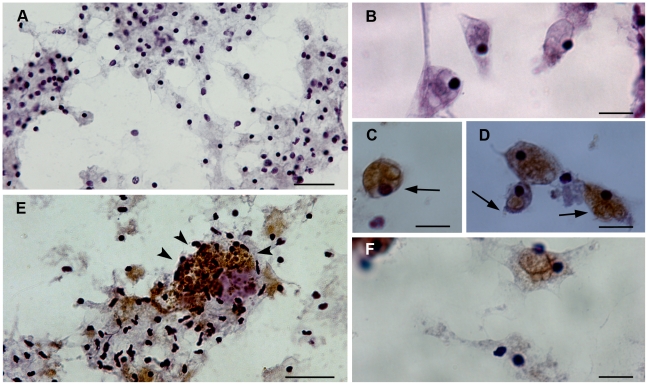
Myt C expression in mussel hemocytes at the protein level. Control hemocytes without anti-Myticin sera but with secondary antibody are presented in figures A and B. A positive reaction is detected by the deposition of brown deposits following DAB treatment (C, D, E and F). Degranulation of hemocytes is indicated with arrowheads. The presence of myticin on vacuoles (at the surface or inside) is indicated with arrows. Scale bars: 50 µm in A and E and 10 µm in B, C, D and F.

Consistent with the Myt C RNA expression pattern, immunocytochemical and immunohistochemical analysis using the sera produced against the two Myt C partial sequence peptides also showed that the hemocytes were the main source of mussel Myt C peptide (Figure 2 and Figure S1, respectively). Notably, the highest expression of Myt C was found in the hemocytes located in the gill *plicae* base (Figure S1). However, according to the RNA and protein expression patterns, Myt C expression seemed limited to some granulocyte subtypes. Regarding the subcellular localization of Myt C, a clear cytoplasmatic expression pattern associated with vacuoles could be observed (Figure 2C, D, F).

### *In vitro* recombinant expression of Myt C variants

**Figure 3:**
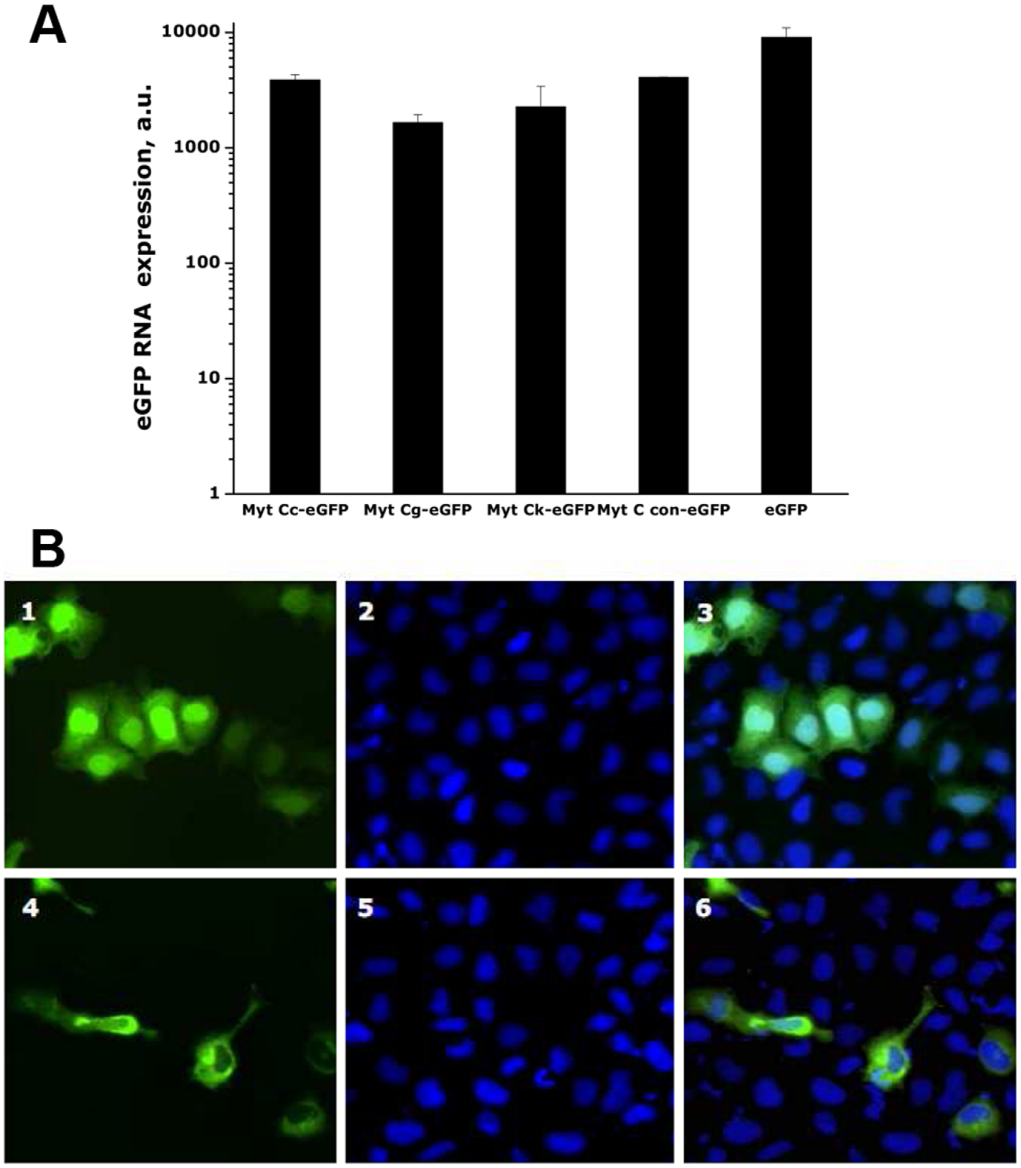
Recombinant expression of Myt C-eGFP variant C in CHSE cells. CHSE cells were transfected with pMCV1.4, pMCV1.4-eGFP or pMCV1.4-Myt Cc-eGFP plasmids, and expression of eGFP and Myt Cc-eGFP was assessed 24 h later at the transcriptional and protein levels. A, expression levels of transcripts of eGFP and Myt Cc-eGFP, Myt Cg-eGFP, Myt Ck-eGFP and Myt Ccon-eGFP evaluated by RT-qPCR using eGFP primers. The data are presented as the mean ± S.D. of two independent experiments, each performed in duplicate. a.u; arbitrary units. B, CHSE expressing eGFP protein (upper panel) or Myt Cc-eGFP fusion protein (lower panel). CHSE micrographs at fluorescent (1,4); UV (2,5); (3,6) merged image of fields 1 and 2.

The levels of expression of the recombinant variants of Myt C (Myt Cc, Myt Cg, Myt Ck and Myt Ccon) were analyzed *in vitro* by RT-qPCR and fluorescence microscopy after transfecting CHSE cells with the corresponding plasmid constructs (Figure 3 and Figure S2).

To evaluate the expression of recombinant Myt C at the transcription level, the presence of cell transcripts containing the eGFP sequence was used as a marker. All Myt C-GFP variants were expressed at comparable levels but slightly lower than the expression of GFP (Figure 3A).

In contrast, to analyze whether the subcellular location of the recombinant Myt C was analogous to that observed in mussel hemocytes *in vivo* (Figure 2), the GFP fluorescence expression pattern was examined by fluorescence microscopy. GFP was diffusely distributed in the cytoplasm and the nucleus of CHSE cells transfected with pMCV 1.4-eGFP (Figure 3B, panel 1 and 3). Addition of the Myt C cDNA sequence to the N-terminus of GFP caused a relocalization of the fluorescence within the cells. Thus, 48 h after transfecting the CHSE cells with plasmids encoding the Myt C variants fused to GFP, the bulk of the fluorescence appeared in the perinuclear region (Figure 3B, panel 4 and 6 and Figure S2) where the trans-Golgi network is usually located. Moreover, nearly all of the cells exhibited a slight non-uniform granulated cytoplasmic distribution of the fluorescence (Figure 3B, panel 4 and 6 and Figure S2, panels 1, 4 and 7) and a peripheral fluorescence pattern near the cellular membrane suggesting the possibility of Myt C extracellular secretion. In contrast with the CHSE cells expressing GFP alone, fluorescence was excluded from the nuclear region in the Myt C-GFP-expressing CHSE cells. We detected no effects of the Myt C expression on CHSE cell morphology or viability, as shown in Figure 3B and Figures S2 and S3. Likewise, those figures show that recombinant Myt C expression did not induce apoptosis.

We observed no differences in the fluorescence pattern among the four variants of Myt C-GFP (Figure S2).

Finally, further confirmation of the cytoplasmic location of recombinant Myt C observed *in vivo* (Figure 2) was obtained by labeling Myt C-GFP expressing cells with the antisera to Myt Cc. As expected, both the GFP fluorescence (Figure S3 panel 1) and immune staining patterns (Figure S3 panel 2) were coincident (Figure S3 panel 4). Antisera to Myt Cc also recognized the Myt C variants k, g and con (not shown).

### Resistance of CHSE cells expressing the Myt C variants to viral infections

**Figure 4:**
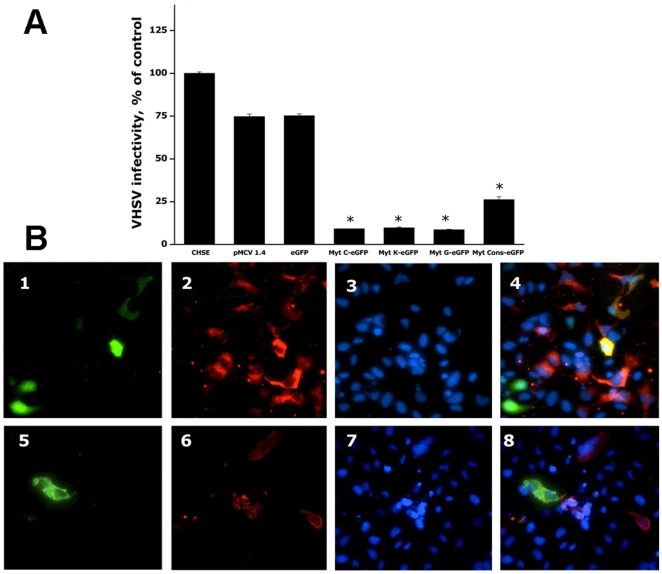
Resistance of CHSE cells expressing the Myt C variants to VHSV infection. CHSE cells were non-transfected or transfected with pMCV1.4, pMCV1.4-eGFP or with each of the pMCV1.4-Myt C-eGFP plasmids for 24 h, washed and then infected with VHSV for 2 h at 14°C. After washing unbound virus, the infected cell monolayers were incubated for 24 h at 14°C, and viral infectivity was estimated by RT-qPCR. A, expression levels of VHSV-pN in infected CHSE cells. The data are presented as the mean ± S.D. from two independent experiments, each performed in triplicate. Asterisks indicate significant differences (p<0.01) in viral infectivity relative to control cells (infected but non-transfected CHSE cells). B, VHSV replication in CHSE-Myt C expressing cells. CHSE cells were transfected with pMCV1.4-eGFP (upper panel) or pMCV1.4-Myt Cc-eGFP (lower panel) plasmids and infected with VHSV as above. Cell monolayers were then fixed and stained. 1 and 5 GFP fluorescence (green fluorescence); 2 and 6, stained with the MAb I10 anti-G protein of VHSV and anti IgG-TRITC (red fluorescence); 3 and 7, stained with the Hoechst DNA stain (blue fluorescence); 4 and 8, merged images.

**Figure 5:**
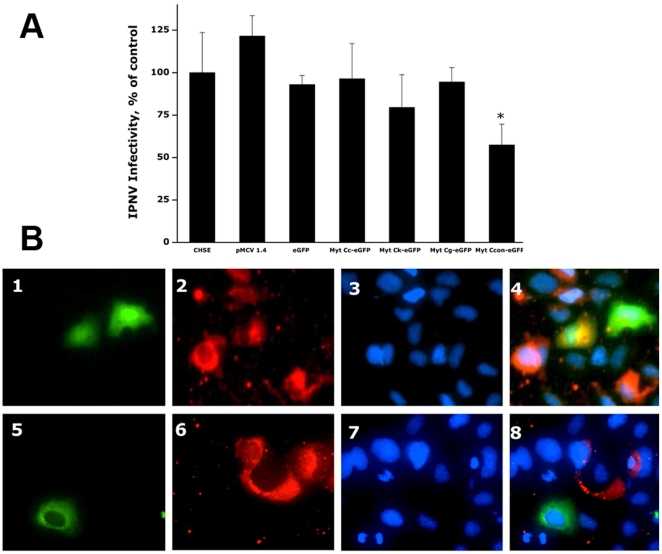
Resistance of CHSE cells expressing the Myt C variants to IPNV infection. CHSE cells were non-transfected or transfected with pMCV1.4, pMCV1.4-eGFP or with each of the pMCV1.4-Myt C-eGFP plasmids for 24 h, washed and then infected with VHSV for 2 h at 14°C. After washing unbound virus, the infected cell monolayers were incubated for 24 h at 16°C, and viral infectivity was estimated by RT-qPCR. A, expression levels of IPNV segment A in infected CHSE cells. The data are presented as the mean ± S.D. from two independent experiments, each performed in triplicate. Asterisks indicate significant differences (p<0.01) in viral infectivity relative to control cells (IPNV infected but non-transfected CHSE cells). B, IPNV replication in CHSE-Myt C expressing cells. CHSE cells were transfected with pMCV1.4-eGFP (upper panel) or pMCV1.4-Myt Cc-eGFP (lower panel) plasmids and infected with IPNV as above. Cell monolayers were then fixed and stained. 1 and 5 GFP fluorescence (green fluorescence); 2 and 6, stained with the MAb 2F12 anti-VP2 protein of IPNV and anti IgG-TRITC (red fluorescence); 3 and 7, stained with the Hoechst DNA stain (blue fluorescence); 4 and 8, merged images.

To carry out the antiviral activity studies, CHSE cells were transiently transfected with each of the plasmids encoding the Myt C variants. Seventy-two hours after transfection, the CHSE cells were infected, and Viral Hemorrhagic Septicaemia virus (VHSV) and Infectious pancreatic necrosis virus (IPNV) infectivity was determined 24 h later. The results showed ≥85% reduced VHSV infectivity in CHSE cells transfected with Myt C variants c, g and k and ∼75% with Myt Ccon (Figure 4A). Control CHSE cells, CHSE cells transfected with empty pMCV 1.4 or pMCV 1.4-eGFP plasmids, propagated the virus almost as efficiently as non-transfected CHSE cells because VHSV infectivity was not significantly reduced (Figure 4A). These data revealed that CHSE cells expressing Myt C had reduced susceptibility to VHSV. In contrast, no significant reduction of IPNV infectivity in CHSE cells expressing Myt C was observed, except in the cells expressing the Myt Ccon (Figure 5A).

Furthermore, viral replication could not be detected in the cells expressing the Myt C variants but was detected in the surrounding non-expressing cells (Figures 4B and 5B, panels 5–8). In contrast, both VHSV (Figure 4B, panels 1–4) and IPNV (Figure 5B, panels 1–4) viruses could be detected in the cells expressing eGFP alone.

### *In vivo* recombinant expression of MytC

**Figure 6:**
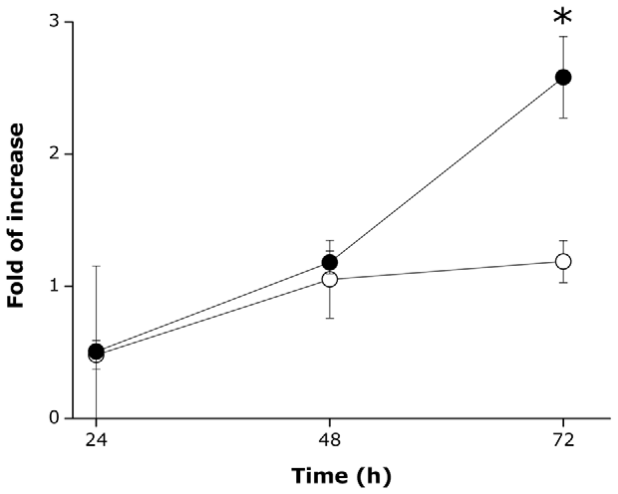
Recombinant expression of Myt Cc-GFP in mussels. Expression of Myt C following injection in adductor muscle of 2.5 µg of pMCV1.4 empty plasmid or pMCV1.4-Myt Ccon-eGFP. Control mussels were injected with an equal volume of PBS. The results are represented as mean ± SD of three different biological replicates in a representative experiment. Asterisks indicate significant differences (p<0.05) in Myt C expression in mussels injected with pMCV1.4 empty plasmid (white circles) or pMCV1.4-Myt Ccon-eGFP (black circles) relative to the expression of PBS injected mussels, previously normalized to 18S transcript levels.

Among the four Myt C variants used in this work, the consensus Myt C (Myt Ccon) was chosen for *in vivo* expression assays because it was the only isoform that inhibited both VHSV and IPNV infectivity *in vitro*. Myt C expression in hemocytes was observed after the plasmid pMCV1.4-Myt Ccon-eGFP was injected into mussel adductor muscle (Figure 6). After 72 h, mussels injected with pMCV1.4-Myt Ccon-eGFP showed a significantly higher hemocytic Myt C expression (p<0.05) compared with the Myt C expression observed in mussels injected with PBS and pMCV1.4 control.

### Expression pattern of immuno-related genes induced by the overexpression of recombinant Myt C *in vivo*

**Figure 7:**
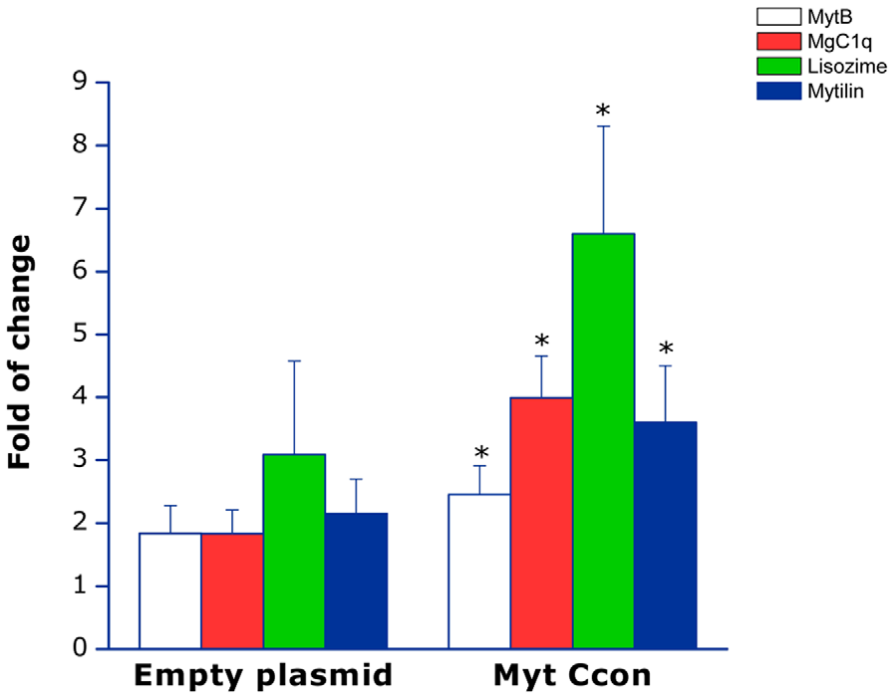
Expression pattern of immune-related genes induced by *in vivo* overexpression of recombinant Myt C. Expression of a selected set of genes related to immune response in mussels. Mussels were injected in adductor muscle with 2.5 µg of pMCV1.4 empty plasmid or pMCV1.4-Myt Ccon-eGFP. Control mussels were injected with an equal volume of PBS. The results represented as the mean ± SEM of three different biological replicates in a representative experiment. Asterisks indicate significant differences (p<0.05) in gene expression in mussels injected with pMCV1.4 empty plasmid or pMCV1.4-Myt Ccon-eGFP relative to the expression of PBS-injected mussels, previously normalized to 18S transcript levels. White bars, *myt b*; red bars, *mgc1q*; green bars, *lysozyme* and blue bars, *mytilin b*.

To investigate the potential immunoregulatory properties of Myt C, the levels of expression of a set of immune-related genes were analyzed by RT-qPCR in the hemocytes of mussels intramuscularly injected with empty pMCV1.4 plasmid or pMCV1.4 Myt Ccon-eGFP. Figure 7 shows the selected immune-related genes that have significantly different levels of expression (p<0.05) compared with controls. Two antimicrobial peptides (mytilin B and myticin B), the C1q domain containing protein (MgC1q) and lysozyme had significantly increased expression at 72 h in animals injected with pMCV1.4 Myt Ccon. The expression of macrophage migration inhibitory factor (MIF) also increased, but not significantly (data not shown). Of note, the expression of Macp and mytimicin (MMG1) also increased after injection with the pMCV1.4 empty plasmid control (data not shown).

### Chemotaxis

**Figure 8:**
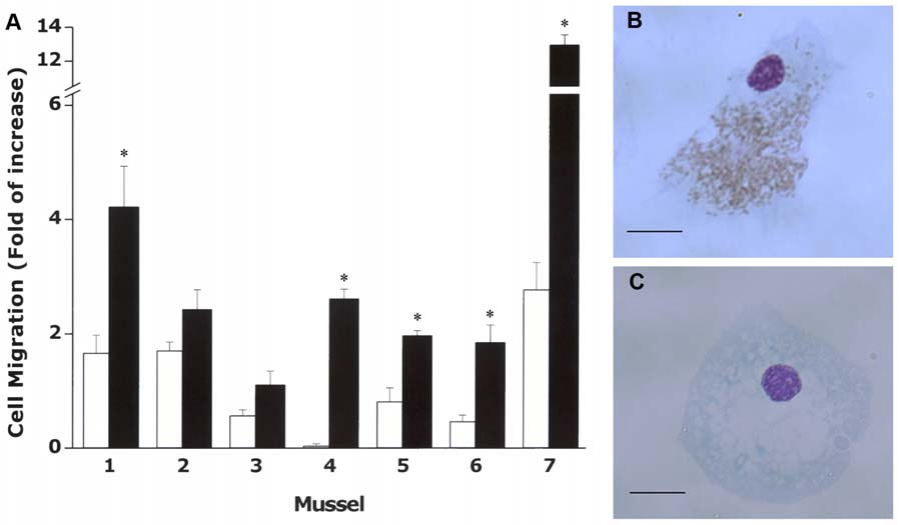
Chemotactic activity of Myt C. Chemotaxis of fresh hemocytes in response to lysates from CHSE transfected cells with pMCV1.4, pMCV1.4-eGFP and pMCV 1.4-Myt Ccon-eGFP vector and CHSE non-transfected cells. Hemocytes from individual mussels were seeded in the upper chamber and lysates from CHSE cells in the lower chamber. A. The number of migrating cells from representative individual mussels to the lower chambers is expressed as the mean ± SD of the cellular counting of five different microscopic fields, relative to hemocytes migrating to lower chambers containing FSW. Asterisks denote statistically significant differences when comparing with CHSE non-transfected cells (p<0.01). B. Aspect of representative hemocytes after migrating to the lower chamber containing pMCV 1.4-Myt Ccon-eGFP. C. Aspect of representative hemocytes after migrating to the lower chamber containing control treatments. Scale Bar: 10 µm.

The recombinant Myt Cc elicited a chemotactic response from hemocytes in the majority of mussels studied (75%). Importantly, there was variability of the magnitude of the chemotactic response among different individual mussels ranging from a 1.1 to a 134-fold increase compared with the migration in control solutions (Figure 8A). Therefore, we have presented values from individual mussels instead of mean ± SD. The hemocytes that migrated through the cell insert to the Myt suspension exhibited a distinct morphology (Figure 8B) compared with hemocyte migration in control suspensions (Figure 8C); contained a higher number of cytoplasmic granules.

## Discussion

Due to the lack of adaptive immunity, AMPs play a key role in the immune system of invertebrate organisms, such as mussels [8], [9], [10]. Thus, mussels and other bivalves can be considered as an interesting source of these innate immunity effector molecules, and due to their wide range of antimicrobial action, AMPs could be used to control infectious diseases that transcend a single aquatic species or pathogen.

It has been recently reported that Mediterranean mussels (*M. galloprovincialis*), that have experienced fewer mass mortalities than other edible bivalves, have an AMP Myt isoform, Myt C, which is highly polymorphic at the mRNA level [11], [12]. Although it has been suggested that the Myt C polymorphism could constitute a molecular adaptation to the interaction of these peptides with the surrounding pathogens [13], the exact role of Myt C has not been clearly established. Therefore, this work was aimed at elucidating the expression pattern of this interesting molecule and the immunological role of Myt C, both as effector and modulator molecule. First, we determined the *in vivo* tissular and cellular Myt C expression patterns (Figures 1 and 2) at the transcriptional and protein levels, using specific RNA probes and antisera to Myt C. Both of the RNA and protein-based analyses showed that Myt C is expressed in hemocytes, consistent with previously described expression of other AMPs in invertebrates for other AMPs [9], [14], [15]. In contrast, Myt C was not expressed in all mussel hemocyte or tissues types, as was previously suggested for defensins and mytilins [15], [16]. Circulating hemocytes expressing Myt C were observed in muscle, connective tissue, gonad and gills by ISH (Figure 1). The strongest expression of Myt C was detected in the *plicae* base of the gills (Figure S1). Because mussels are filter-feeding animals that can inhabit polluted locations and even feed on bacteria [17], [18], the gills are the first tissue to come in contact with putative pathogens; the high presence of Myt C-expressing hemocytes could suggest a role for this AMP in a fast immune response.

Next, to investigate the potential antimicrobial activity of Myt C, we cloned and expressed eGFP fusions of the four different cDNA sequences of Myt C, including the previously determined consensus sequence of the Myt C gene, *in vitro*. The complete sequence of the Myt C prepropeptide was included in the plasmids used to transfect the CHSE cells to assure that all of the information required to process mature Myt C was present. The levels of Myt C expression in transfected CHSE cells were quite similar at both the RNA (Figure 3A) and protein levels for all of the constructs studied (Figure 3B and Figure S2). However, visualisation of the intracellular GFP fluorescence in living CHSE cells expressing eGFP or the different Myt C-eGFP variants (Figure 3B and Figure S2) showed that eGFP was homogeneously present throughout the entire cell, but Myt C-eGFP had a different subcellular localization. Overall, Myt C-eGFP was dispersed throughout the cytoplasm of the cell with a slightly granular appearance. This granular appearance of fluorescence suggests the accumulation of the fusion protein inside vesicular structures along the secretory pathway. This result is consistent with the *in vivo* Myt C expression results (Figure 1 and 2) and previous reports that mussel AMPs, defensin and mytilin, are localized to cytoplasmatic granules [15], [19]. The addition of eGFP to the C-terminus of Myt C did not seem to modify the cellular expression pattern of this AMP; this result was confirmed when Myt C-expressing cells were stained with antisera to Myt C (Figure S3).

Because some reports have shown that AMPs possess antiviral activity against enveloped and non-enveloped viruses [20], [21], with some of them acting across species [22], [23], the antiviral activity of recombinant Myt C was studied against the fish enveloped and non-enveloped viruses, VHSV and IPNV, respectively. The results clearly showed that Myt C could be considered as an invertebrate antiviral immune effector, at least against the fish viruses that were tested in this study. Notably, Myt C-transfected cells were more resistant to VHSV infection than to IPNV infection, regardless of the Myt C variant expressed by the cells (Figure 4A). In fact, only the Myt Ccon variant was able to confer protection against IPNV (Figure 4B). Therefore, enveloped viruses seem to be more susceptible to Myt C because of the potential ability of this AMP to disrupt lipid membranes, as it has been previously described for other mussel AMPs, such as defensin and mytilin [10], [24].

Furthermore, no viral replication could be observed in the CHSE Myt C expressing cells (Figure 5), suggesting that Myt C expression helped these cells to overcome viral infection. Moreover, very low levels of VHSV replication could be detected in the CHSE Myt C-expressing cells compared with the eGFP-transfected or non-transfected cells, suggesting the induction of some protective effect on the surrounding cells by the expression of Myt C, which could act as a cytokine-like molecule. Alternatively, Myt C could be secreted outside the expressing cells to inactivate the virus particles released from the initially infected cells. Work is in progress to clarify the mechanism underlying the antiviral activity of mussel Myt C.

In an attempt to investigate the potential immunomodulatory properties of Myt C, mussels were transfected with a plasmid encoding the Myt Ccon variant, and the gene expression patterns of several immune related genes were analyzed. It cannot be ignored that the functionality of this consensus sequence could not be 100%. Further research will detail the generation of this variability and determine the relative importance of the different isoforms in pathogen recognition and modulation of the immune response. However, Myt Ccon was the only isoform that was completely functional in antiviral experiments with the two viruses analyzed in this study.

The overexpression of Myt Ccon induced significant changes in the expression of Myticin B, MgC1q, Lysozyme and Mytilin B. Myticin B is another member of the mussel myticin group that is constitutively expressed in mussel hemocytes [25]. Myticin B is expressed in hemocytes in virtually all mussel tissues and has antibacterial activity against Gram-positive bacteria [15], [26]. Mytilin B is a distinct antimicrobial peptide that is constitutively expressed in adult mussels, and a decrease in the expression of this gene is observed after bacterial challenge [8]. MgC1q is a complement C1q domain-containing protein, recently characterized in *M. galloprovincialis,* which is highly induced after Gram-positive or Gram -negative bacterial challenge [27]. MgC1q seems to have the same properties as a pattern recognition receptor [28], [29]. Lysozyme is a well-studied, marine invertebrate effector molecule able to kill Gram-negative bacteria and also with antifungal properties [30], [31], [32]. These data suggest that Myt C not only has a role in the antiviral activities of mussels but could also have modulator effects over different molecules: activating and triggering the mussel innate immunity system both at recognizing (MgC1q) and effector (lysozyme, myticin B) expression levels. Non-specific changes in Macp and MMG1 expression could be explained by unmethylated CpG motifs present in the vector DNA, indicating that mussels are able to mount an immune response against bacterial DNA, such as has been described both in vertebrates and invertebrates [33], [34], [35].

Lysates from Myticin C-transfected cells induced significant cell migration in individual mussels as compared with the lysates from different controls. Chemotactic effects play a central role in the inflammation process because they elicit the migration of cells to the site of injury. Triggering chemotaxis is usually an important mechanism for pathogen recognition by immune cells [36], [37]. Molecules, such as opioid neuropeptides or TGF-β1 and PDGF-AB, have shown chemotactic activity in *Mytilus edulis* [38], [39]. In the gastropod, *Clithon retropictus,* chemotaxis has been described as a more efficient process in adult animals than in juveniles [36]. Myt C is highly expressed in oocytes but is not expressed in the spermatozoids of *M. galloprovincialis* [11], suggesting that this chemotaxis could also have a role in reproduction. Hemocytes chemotactically-induced by Myt C have abundant granules present in the cytoplasm (Figure 8B) and have a distinct morphology compared with treated control hemocytes, suggesting an activated status. Although there are no descriptions of homologous cytokines or chemokines in invertebrates, Myt C could play the role of mediating immune activation because it induces migration of particular hemocyte subtypes and triggers hemocyte gene expression.

The high degree of nucleotide variability of Myt C sequences has elicited questions regarding their function since they were first described [12], [13]. Here, we have studied expression of this AMP and looked for antiviral, chemotactic and immunoregulatory properties. Expression analysis at both the mRNA and protein levels revealed that hemocytes were the main Myt C-expressing cell type. Transfection analysis in the fish cell line, CHSE, revealed that different isoforms of Myt C were mainly active against the enveloped virus, VHSV, which is an important fish virus that causes massive mortalities. In addition, transfection also revealed that the cytomegalomovirus early promoter could direct the *in vivo* expression of Myt C in mussels, resulting in an increase in the expression of other immune-related genes in mussel hemocytes. Recombinant Myt C also showed chemotactic activities in mussel hemocytes. Together with the induction of the expression of effector molecules, such as Myt B, Mytilin B and Lysozyme, this data suggests its role as a cytokine in the mussel immune response.

Altogether, our results suggest that Myticin C might influence the outcome of infection in the following ways: a) acting as direct antiviral molecule, particularly against enveloped viruses, b) inducing hemocyte chemotaxis and c) modulating the expression of other immune genes that carry out and probably amplify the mussel immune response.

## Materials and Methods

### Animals

Mediterranean mussels (*M. galloprovincialis*) were obtained during low spring tide from wild populations. Animals were kept in an open aerated seawater system at 15°C and fed daily with a mixture of the phytoplanktonic species *Isochrysys galbana*, *Tetraselmis svecica* and *Skeletonema costatum*. Animals were acclimatized for at least one week prior to the experiments. Molluscs care and experiments were conducted according the CSIC National Committee on Bioethics guidelines under approval number ID 20.03.11.

### *In situ* hybridization

*In situ* hybridization (ISH) was carried out to localize the expression of the myticin gene, as described in Murray et al. [40] with minor modifications.

#### *Probe preparation*

Digoxigenin-labeled Myt C-specific RNA probes, antisense (As) and sense (S; control) (GenBank Accession Number JF323020), were prepared by *in vitro* transcription of cDNA clones using the DIG RNA labeling kit (SP6/T7) (Roche Diagnostics, Mannheim, Germany) according to the manufacturer's protocol.

#### Tissue *in situ* hybridization

Dorsoventral mussel sections of 0.5 cm were fixed overnight at 4°C in 4% formalin solution and manually dehydrated through an ethanol series, cleared in xylene and embedded in paraffin for sectioning. Serial sections of 7 µm were cut from the block and placed on polylysine-coated glass slides (Thermo Scientific, Waltham, MA, USA), briefly air dried and baked overnight at 60°C.

The ISH assays were performed simultaneously on two sets (As and S) of duplicate slides, each one with serial sections from the same animal. Briefly, deparaffinized, rehydrated tissue sections equilibrated in fish saline [41] were pre-digested with proteinase K (2.5 µg ml^−1^) and treated with the probes (50 µl) diluted with *in situ* hybridization buffer at 47°C overnight. Slides were subsequently incubated with RNase A and RNase T1 buffer for 30 min at 37°C to eliminate non-hybridized probes and washed in saline sodium citrate buffer (SSC). After incubation with blocking buffer, digoxigenin was detected using sheep anti-DIG-alkaline phosphatase-conjugated antibodies (1∶250) (Roche Diagnostics, Mannheim, Germany). Alkaline phosphatase was detected using NBT (nitroblue tetrazolium)/BCIP (5-bromo-4-chloro-3-indolyl-phosphate) (Roche Diagnostics, Mannheim, Germany).

#### Hemocyte *in situ* hybridization

Detection of Myt C expression in hemocytes was conducted with the probes synthesized as described above. The hemocytes were collected and immediately fixed in 5 volumes of 4% formalin solution. After washing in 70% ethanol, hemocytes were placed in two sets (As and S) of duplicate polylysine slides (Thermo Scientific, Waltham, MA, USA) and centrifuged at 750 rpm for 5 min using a cytocentrifuge (Cytospin 4 Cytocentrifuge, Thermo Scientific, Waltham, MA, USA). Slides were washed with PBS for 10 min, and hemocytes were permeabilized with proteinase K (4 µg ml^−1^) at 37°C for 10 min. Hemocytes were then fixed in 4% cold formaldehyde (10 min at 4°C), rinsed with 2X SSC and treated with hybridization buffer for 90 min at 37°C in a humid incubation chamber. Probes (50 ng per slide) were added to slides and incubated overnight at 37°C on gene frames (Abgene, Epsom, UK). After washing, digoxigenin was detected as explained above.

### Synthetic peptides from the sequence of Myt C cDNA

*In silico* translation (http://www.expasy.org) from the cDNA sequence of a Myt C variant prepropeptide (Tables 1 and 2), now called Myt Cc, was used for the synthesis of 13- and-16-mer peptides, p13 and p16 (Table 1), respectively. Synthesis was performed by New England Peptide (Gardner, MA, USA) with a purity of >95%, as determined by high-performance liquid chromatography and mass spectrophotometry. Peptides were reconstituted to a final concentration of 1 mg ml^−1^ in Milli Q water and stored in suitable aliquots at −80°C until used.

**Table 1:** Sequence and relative position of synthetic peptides used in present study.

Peptide name	Sequence to C-terminal	Position (From aa to aa)
Myt Cc prepropeptide	MKATILLAVVVAVIVGVQEAQSVACRSYYCSKFCGSAGCSLYGCYLLHPGKICYCLHCSRAESPLALSGSARNVNDKNNEMDNSPVMNEMENLDQEMDMF	1–100
p13	HPGKICYCLHCSR	48–60
p16	CSARNVNDKNNEMDNS	69–84
Mature Myt Cc	QSVACRSYYCSKFCGSAGCSLYGCYLLHPGKICYCLHCSR	20–60

**Table 2:** Sequences of primers and probes for RT-qPCR.

Target	Primer Name	Sequence (5′>3′)	Probe	Reference (accession number)
Enhanced Green Fluorescent Protein	*egfp fw*	ATGGTGAGCAAGGGCGAGGAG		[49]
	*egfp rv*	CCGCTTTACTTG TACAGCTCG		
Protein N of VHSV	*N_VHSV_ fw*	GACTCAACGGGACAGGAATGA	TGGGTTGTTCACCCAGGCCGC	[54]
	*N_VHSV_ rv*	GGGCAATGCCCAAGTTGTT		
Segment A of IPNV	*IPNV A segment fw*	TCTCCCGGGCAGTTCAAGT	CCAGAACCAGGTGACGAGTATGAGGACTACAT	[55]
	*IPNV A segment rv*	CGGTTTCACGATGGGTTGTT		
Myticin Cc	*myt Cc fw*	ATTTGCTACTGCCTTCATTG		Present Work
	*myt C rv*	TCCATCTCGTTGTTCTTGTC		
Elongation factor 1 α	*ef1-α fw*	ACCCTCCTCTTGGTCGTTTC	GCTGTGCGTGACATGAGGCA	[56]
	*ef1-α rv*	TGATGACACCAACAGCAACA		
Mytimycin Precursor 1	*MMG1 Mytimycin Prec qPCR1 S*	ACGGATGACGCTTTTGTTTG		Present Work (FJ804479)
	*MMG1 Mytimycin Prec qPCR1 As*	GCAGTCCCAGCAATGTTTC		
Macrophage migration inhibitory factor	*MIF qPCR1 S*	TACACCCAGACCAAATGATG		Present Work (FL498330)
	*MIF qPCR 1 As*	TTCTCCTAATGCTCCAATACTG		
Myticin B	*Myticin B qPCR 1S*	AATGTCTTCGTTGTTCCAG		Present work (AF162336)
	*Myticin B qPCR 1As*	AATGCCAGTTTCACCTTG		
MgC1q	*MgC1q qPCR 1S*	ATTTATGCGTTCACTTGGAC		Present Work (FN563147)
	*MgC1q qPCR 1As*	ACACCGATTTTTGTGCTG		
Lysozyme	*Mg Lysozyme qPCR 1S*	TGTCTGTCGCACTATTCTTC		Present Work (AF334665)
	*Mg Lysozyme qPCR 1As*	AGTCCGCAACAAACATTC		
Mytilin B	*Myt 4*	TGAAGGCAGGAGTTATTCTGGC		[8]
	*Myt 3*	ACAACGAAGACATTTGCAGTAGC		
Macp	*Macp PCRq-F*	AAGGTGGATGTTGGTTATGGAGAA		Present Work (HQ709239)
	*Macp PCRq-R*	GCCCAATCAGGCATCATGTTA		

### Production of antiserum to Myt C in the rabbit

To obtain the antisera (New England Peptide, Gardner, MA, USA), rabbits were first co-immunized with 1 mg ml^−1^ of each of the synthetic peptides, p13 and p16 (Table 1), and diluted 1∶1 in Freund's complete adjuvant. Four weeks later, a second injection with the same antigens in Freund's incomplete adjuvant were given. Blood was collected before injection (pre-immune serum) and at 30 days after the second injection. The collected blood sample was subsequently incubated for 2 hours (h) at 4°C and centrifuged to obtain the serum. Finally, affinity purification of antisera over each respective peptide column was carried out to obtain two antisera with distinct immunoreactivity: anti-Myt C p13 detected the Myt Cc mature peptide, and anti-Myt C p16 detected the Myt Cc propeptide sequence (Table 1).

### Immunodetection of Myticin C

Immunohistochemical staining was performed using a mixture (1∶1) of the affinity purified antisera, anti-Myt Cc p13 and p16 on paraffin embedded mussels fixed in Davidson's fixative. After deparaffination, peroxidase activity was suppressed with 3% hydrogen peroxide in methanol for 4 min. Antigen exposition was achieved using proteinase K treatment (20 µg ml^−1^) for 10 min. A blocking step was carried out in 1% bovine serum albumin (BSA)-containing Tris-Buffered Saline with Tween-20 (TBST) for 1 h at room temperature. Then the mixture of antisera to Myt Cc diluted 50-fold in 1% BSA-containing phosphate buffered saline (PBS) was applied to the slides and incubated overnight at 4°C. Myt C was detected by incubating the slides with a peroxidase-conjugated goat anti-IgG rabbit antibody (Sigma Chem. Co, St. Louis, MO, USA) diluted 500-fold in 1% BSA-containing PBS for 2 h at room temperature. The peroxidase activity was detected using *SIGMAFAST™* Diaminobenzidine (DAB) tablets (Sigma Chem. Co, St. Louis, MO, USA). All of the washing steps were conducted with PBS. Finally, the slides were lightly counterstained with hematoxylin for 5 s and mounted in Permount Slide Mounting Fluid.

To carry out the immunocytochemistry staining, 100 µl of mussel hemolymph were centrifuged (750 rpm, 5 min) in a cytocentrifuge (Cytospin 4 Cytocentrifuge, Thermo Scientific, Waltham, MA, USA). After acetone fixation, blocking and immunostaining were carried out as indicated above.

The photographs from the previous sections (*in situ* hybridization and immunodetection) were obtained with a DXM 1200 digital camera mounted on a Nikon Eclipse 80i light microscope (Nikon instruments Inc., NY, USA).

### Constructs and plasmids

The plasmids used were pMCV 1.4 (Ready-Vector, Madrid, Spain) [42] and pGFP, which is 3.4 kbp in length (Clontech, Mountain View, CA, USA) and contains the eGFP cDNA sequence under the control of the cytomegalovirus early promoter (CMV).

To obtain the pMCV1.4-Myt C constructs, several Myt C cDNA sequences were synthesized at Biost (Montreal, Canada). The variants are Myt Cc, Myt Cg, Myt Ck and Myt Ccon (Table S1). Of note, the sequence of the so-called variant Myt Ccon is a previously established consensus sequence of the Myt C gene [12]. Each of these synthetic nucleotide sequences was then cloned into the pMCV1.4 plasmid digested with the restriction enzymes KpnI and XbaI following standard procedures. To obtain the different pMCV1.4-Myt C-eGFP constructs, the eGFP cDNA sequence was first excised from the pGFP plasmid with the restriction enzymes XbaI and BamHI and then subcloned into each of the pMCV1.4-Myt C plasmids digested with the same enzymes. The products were resolved on a 1% agarose gel, and the DNA bands were extracted from the gel and purified using GeneClean (Bio 101, Vista, CA, USA).

### Cell cultures and virus

The fish cell lines used in this work, CHSE-214 (Chinook salmon embryo) and EPC (Epithelioma papulosum cyprinid), were purchased from the American Type Culture Collection (ATCC numbers CRL-1681 and CRL-2872, respectively). Both cell lines were maintained at 20°C in a 5% CO_2_ atmosphere with RPMI-1640 Dutch modified cell culture medium (Gibco, Invitrogen Co., Carlsbad, CA, USA) containing 10% fetal calf serum (FCS) (Sigma Chem. Co, St. Louis, MO, USA), 1 mM pyruvate (Gibco, Invitrogen Co., Carlsbad, CA, USA), 2 mM glutamine (Gibco, Invitrogen Co., Carlsbad, CA, USA), 50 µg ml^−1^ gentamicin (Gibco, Invitrogen Co., Carlsbad, CA, USA) and 2 µg ml^−1^ fungizone (Gibco, Invitrogen Co., Carlsbad, CA, USA).

Viral hemorrhagic septicemia virus strain 07.71 (VHSV_07.71_) was isolated in France from rainbow trout (*Oncorhynchus mykiss*) [43] and propagated in the EPC cell line at 14°C as previously reported [44]. Infectious necrosis pancreatic virus (IPNV) serotype sp., purchased from the American Type Culture Collection (ATCC number VR-1318), was propagated in the CHSE-214 cell line at 16°C as previously described [45]. In both cases, supernatants from infected cell monolayers were clarified by centrifugation at 4000 g during 30 min and kept in aliquots at −70°C. Clarified supernatants were used for the experiments. The VHSV and IPNV stocks were titrated in 96-well plates using a previously developed immunostaining focus assay (focus forming units, f.f.u.) [22], [46], [47] and the end-point dilution method [48], respectively.

### Transfection assays

CHSE-214 cells were transfected with the empty plasmid pMCV 1.4 or with plasmids encoding eGFP or the different Myt C-eGFP sequences. Cell transfections were carried out as described previously [49], [50], [51]. Briefly, CHSE-214 monolayers were detached using TrypLE™ Select (Gibco, Invitrogen Co., Carlsbad, CA, USA), resuspended in RPMI-1640 Dutch-modified cell culture medium with 10% FBS and dispensed into 96-well plates (4×10^4^ cells per well) in a final volume of 100 µl. Then, 0.2 µg of each plasmid, complexed with 0.3 µl of FuGene HD (Roche Diagnostics, Mannheim, Germany), were added to each well, and the plates were incubated at 20°C for 3 days. Myt C-eGFP-transfected cells were viewed and photographed with an inverted fluorescence microscope (Nikon Eclipse TE2000-U, Nikon instruments Inc., NY, USA) provided with a digital camera (Nikon DS-1QM).

### Analysis of Myt C expression in transfected cells

The expression of Myt C variants in CHSE transfected cells was analyzed at both the transcriptional and protein levels by quantitative real time RT-PCR (RT-qPCR) and immunofluorescence (IF), respectively.

Because Myt C variants were expressed as fusion proteins with eGFP, the expression of eGFP was used as marker to evaluate their transcriptional levels. RT-qPCR was used to evaluate the expression levels of the eGFP transcripts using eGFP cDNA-specific primers (Table 2) and SYBR Green (Applied Biosystems, Foster City, CA, USA).

To detect Myt C peptides, CHSE cells expressing Myt C-eGFP fusion proteins were grown in 96–well plates, fixed with BD Cytofix (BD Biosciences, Franklin Lakes, NJ, USA) for 15 min at room temperature and permeabilized with 0.2% Triton X100 (Merck, Darmstadt, Germany) for 5 min at room temperature. Cell monolayers were then incubated with the mixture (1∶1) of the affinity purified sera anti-Myt Cc diluted 300-fold in 0.1% BSA-containing PBS for 2.5 h at room temperature. Using goat anti-rabbit antibody conjugated to rhodamine (TRITC, Sigma Chem. Co, St. Louis, MO, USA), the indirect staining was carried out. Finally, to visualize cell nuclei, cell monolayers were incubated with 0.1 mg ml^−1^ of the Hoechst DNA stain (Sigma Chem. Co, St. Louis, MO, USA) for 10 min. Immunostained cells were viewed and photographed with an inverted fluorescence microscope (Nikon Eclipse TE2000-U, Nikon instruments Inc., NY, USA) provided with a digital camera (Nikon DS-1QM).

### Viral infection assays

Transfected CHSE cells were washed extensively with PBS and infected with VHSV or IPNV (multiplicity of infection, m.o.i. of 2×10^−2^) in a final volume of 100 µl/well of culture medium supplemented with 2% FCS for 2 h at 14°C. The infected cell monolayers were then washed, fresh medium was added, and plates were further incubated for 24 h. Viral replication in CHSE cells was evaluated by RT-qPCR using the specific primer and probe sequences for the gene encoding the N protein of VHSV or for the segment A of the IPNV genome (Table 2). Non-transfected CHSE cells that were infected with VHSV or IPNV were included as control.

### Overexpression of Myt C in mussels and gene expression analysis

Mussels were injected in the adductor muscle with either PBS or with 2.5 µg ml^−1^ of empty plasmid pMCV1.4 or pMCV1.4-Myt Ccon plasmids in a volume of 50 µl. Twenty four, 48 and 72 h post-injection, hemolymph was withdrawn from the adductor muscle of each animal with a disposable syringe, and hemocytes were collected by centrifugation at 12000 g for 10 min and subjected to subsequent RNA extraction.

### Chemotaxis assay

Chemotactic properties of the recombinant Myt C were determined using PET cell culture inserts of 8.0 µm pore size (Becton & Dickinson, Franklin Lakes, NJ, USA) in 24-well plates. Briefly, 250 µl of hemolymph from individual mussels (n = 12) was added to the upper compartment, and 400 µl of dilutions of cell extracts were located in the lower compartment. These extracts contained the cellular lysates of CHSE cells transfected with pMCV1.4, pMCV1.4-GFP or pMCV1.4-Myt Cc-eGFP, non transfected CHSE or filtered seawater (FSW). To obtain the extracts, the cells were resuspended in FSW, frozen, thawed and then centrifuged to eliminate cell debris. After 4 h of incubation in the dark at 15°C, cells in the lower compartment were recovered, subjected to cytocentrifugation as described above and stained using the Hemacolor kit (Merck, Darmstadt, Germany) according to the manufacturer's instructions. Cells in the lower chamber were counted using a Nikon Eclipse 80i light microscope.

### RNA isolation and cDNA synthesis

The RNeasy Plus Mini Kit (Qiagen, Hilden, Germany) was used to extract total RNA from the CHSE cells following the manufacturer's instructions. The synthesis of cDNA from 1 µg of RNA, as estimated by a NanoDrop spectrophotometer 200 c (Thermo Scientific, Waltham, MA, USA), was carried out using M-MLV reverse transcriptase (Invitrogen Co., Carlsbad, CA, USA) as previously described [22].

RNA from mussel cells was extracted with Trizol (Invitrogen Co., Carlsbad, CA, USA) according to manufacturer's instructions. Contaminating genomic DNA was removed using DNAse I (Ambion, Applied Biosystems, Foster City, CA, USA). The synthesis of cDNA from 1 µg of RNA was carried out using the SuperScript® III First-Strand Synthesis SuperMix for qRT-PCR (Invitrogen Co., Carlsbad, CA, USA).

### Real Time Quantitative PCR assays

Quantitative PCR in real time (RT-qPCR) was carried out as previously described [22]. All reactions were performed in a 20-µl volume containing 2 µl of the cDNA reaction, 900 nM of each primer, 200 nM of probe and 10 µl of TaqMan Universal PCR Master Mix (Applied Biosystems, Foster City, CA, USA). The cycling conditions were 50°C for 2 min and 95°C for 10 min followed by 40 cycles of 95°C for 15 s and 60°C for 1 min. Gene expression results were analyzed by the 2^−ΔΔCt^ method [52].

To evaluate the eGFP expression, the cellular elongation factor 1 alpha (EF1-α) gene (Table 2) was used as an endogenous control for quantification. The control cells (non-transfected CHSE group) served as the calibrator cells, and fold increases were calculated relative to the level for these cells.

To evaluate virus replication, the internal reference for normalization of data was the cellular 18S rRNA (Applied Biosystems, Foster City, CA, USA). Viral infectivity results were expressed as percentages of infectivity and calculated using the formula (viral infectivity in transfected cells/viral infectivity in non-transfected cells)×100.

To detect gene expression in mussel hemocytes, specific PCR primers (Table 2) were designed and checked for hairpin and dimer formation according to known RT-qPCR restrictions (PCR product size, Tm difference between primers, GC content and self-dimer or cross-dimer formation) with the Oligo Analyzer program, version 1.0.2 (T. Kuulasma, University of Kuopio, Kuopio, Finland, http://molbiol-tools.ca/OASetup102.exe). Then, the efficiency of the primer pairs was analyzed with seven, five-fold serial dilutions of cDNA and calculated from the slope of the regression line of Cts versus the relative concentration of cDNA [53]. A melting curve analysis was also performed to verify that no primer dimers were amplified. If these conditions were not accomplished, new primer pairs were designed. Mytimycin precursor 1 (MMG1), Macrophage migration inhibitory factor (MIF), Myticin B, *Mytilus galloprovincialis* C1q domain containing protein (MgC1q), Lysozyme, Mytilin B and membrane attack complex protein (Macp) were investigated for changes in gene expression. One microliter of 10-fold diluted cDNA template was mixed with 0.5 µl of each primer (10 µM) and 12.5 µl of SYBR green PCR master mix (Applied Biosystems, Foster City, CA, USA) in a final volume of 25 µl. The standard cycling conditions were 95°C for 10 min, followed by 40 cycles of 95°C for 15 s and 60°C for 1 min. All reactions were carried out as technical triplicates. The relative expression levels of the genes were normalized using the 18S gene as a housekeeping gene, which was constitutively expressed and not affected by Myt C overexpression, following the Pfaffl method [53]. Fold change units were calculated by dividing the normalized expression values of the hemocytes from pMCV 1.4 empty plasmid or the pMCV1.4-Myt Ccon-GFP injected mussels by the normalized expression values of the controls.

### Statistical analysis

To analyze the viral infectivity results, statistical comparisons were made using a paired, two-tailed Student *t* test. To analyze differences in gene expression among mussels injected with pMCV1.4, pMCV1.4-Myt or PBS, statistical comparisons were made using a one-tailed Student t-test considering groups of equal variance. The results were expressed as mean ± SEM (square error of the mean), and differences were considered statistically significant when p<0.05. Statistically significant differences in chemotactic properties were determined using a one-tailed Student t-test considering groups of equal variance against control groups. Differences were considered statistically significant when p<0.01.

## Supporting Information

Figure S1 **Immunohistochemical determination of the expression pattern of Myt C in gills.** Positive hybridization is detected by brown deposits following DAB treatment in A (arrowheads). Control tissues not hybridized with anti-Myticin sera are presented in figure B. Scale bars: 25 µm.
(TIF)

Figure S2 **Recombinant expression of Myt C-eGFP variants in CHSE cells as eGFP fusion proteins.** CHSE cells were transfected with pMCV1.4-Myt Cg-eGFP, pMCV1.4-Myt Ck-eGFP or pMCV1.4-Myt Ccon-eGFP plasmids and assessed 24 h later. CHSE micrographs with fluorescent (1, 4 and 7) and UV light (2, 5 and 8); merged image of fields 1 and 2 (3), 4 and 5 (6) and 7 and 8 (9), respectively.
(TIF)

Figure S3 ***In vitro*** **determination of the subcellular localization of recombinant Myt Cc.** Description of data: CHSE cells were transfected with pMCV1.4-Myt Cc-eGFP plasmid and 24 h post transfection washed, fixed and stained with an antiserum anti-Myt C (1), GFP (2) Rho (3) UV (4) merged image of fields 1, 2 and 3.
(TIF)

Table S1 Nucleotide sequences of Myt C variants and the antisense ISH RNA cDNA template used in this study.
(DOCX)

## References

[1] Falcó A, Brocal I, Pérez L, Coll JM, Estepa A (2008). *In vivo* modulation of the rainbow trout (*Oncorhynchus mykiss*) immune response by the human alpha defensin 1, HNP1.. Fish Shellfish Immunol.

[2] Patrzykat A, Douglas SE (2005). Antimicrobial peptides: Cooperative approaches to protection.. Protein Peptide Lett.

[3] Zasloff M (2002). Antimicrobial peptides of multicellular organisms.. Nature.

[4] Oppenheim JJ, Biragyn A, Kwak LW, Yang D (2003). Roles of antimicrobial peptides such as defensins in innate and adaptive immunity.. Ann Rheum Dis.

[5] Klotman ME, Chang TL (2006). Defensins in innate antiviral immunity.. Nat Rev Immunol.

[6] Ulvatne H, Haukland HH, Samuelsen Ø, Krämer M, Vorland LH (2002). Proteases in *Escherichia coli* and *Staphylococcus aureus* confer reduced susceptibility to lactoferricin B.. J Antimicrob Chemother.

[7] Brown KL, Hancock RE (2006). Cationic host defense (antimicrobial) peptides.. Curr Opin Immunol.

[8] Mitta G, Hubert F, Dyrynda EA, Boudry P, Roch P (2000). Mytilin B and MGD2, two antimicrobial peptides of marine mussels: gene structure and expression analysis.. Dev Comp Immunol.

[9] Mitta G, Hubert F, Noel T, Roch P (1999). Myticin, a novel cysteine-rich antimicrobial peptide isolated from haemocytes and plasma of the mussel *Mytilus galloprovincialis*.. Eur J Biochem.

[10] Roch P, Yang Y, Toubiana M, Aumelas A (2008). NMR structure of mussel mytilin, and antiviral-antibacterial activities of derived synthetic peptides.. Dev Comp Immunol.

[11] Costa MM, Dios S, Alonso-Gutiérrez J, Romero A, Novoa B (2009). Evidence of high individual diversity on myticin C in mussel (*Mytilus galloprovincialis*).. Dev Comp Immunol.

[12] Pallavicini A, Costa MM, Gestal C, Dreos R, Figueras A (2008). High sequence variability of myticin transcripts in hemocytes of immune-stimulated mussels suggests ancient host-pathogen interactions.. Dev Comp Immunol.

[13] Padhi A, Verghese B (2008). Molecular diversity and evolution of myticin-C antimicrobial peptide variants in the Mediterranean mussel, *Mytilus galloprovincialis*.. Peptides.

[14] Battison AL (2008). Isolation and characterisation of two antimicrobial peptides from haemocytes of the American lobster *Homarus americanus*.. Fish Shellfish Immunol.

[15] Mitta G, Vandenbulcke F, Noel T, Romestand B, Beauvillain JC (2000). Differential distribution and defence involvement of antimicrobial peptides in mussel.. J Cell Sci.

[16] Mitta G, Vandenbulcke F, Hubert F, Salzet M, Roch P (2000). Involvement of mytilins in mussel antimicrobial defense.. J Biol Chem.

[17] Govorin I (2000). Role of bivalves in the depuration of seawaters contaminated by bacteria.. Russ J Mar Biol.

[18] Birkbeck TH, McHenery JG (1982). Degradation of bacteria by *Mytilus edulis*.. Mar Biol.

[19] Mitta G, Vandenbulcke F, Hubert F, Roch P (1999). Mussel defensins are synthesised and processed in granulocytes then released into the plasma after bacterial challenge.. J Cell Sci.

[20] Hancock REW, Sahl H-G (2006). Antimicrobial and host-defense peptides as new anti-infective therapeutic strategies.. Nat Biotechnol.

[21] Ding J, Chou YY, Chang TL (2009). Defensins in viral infections.. J Innate Immun.

[22] Falcó A, Mas V, Tafalla C, Pérez L, Coll JM (2007). Dual antiviral activity of human alpha-defensin-1 against viral haemorrhagic septicaemia rhabdovirus (VHSV): Inactivation of virus particles and induction of a type I interferon-related response.. Antiviral Res.

[23] Falcó A, Ortega-Villaizan M, Chico V, Brocal I, Perez L (2009). Antimicrobial peptides as model molecules for the development of novel antiviral agents in aquaculture.. Mini Rev Med Chem.

[24] Roch P, Beschin A, Bernard E (2004). Antiprotozoan and antiviral activities of non-cytotoxic truncated and variant analogues of mussel defensin.. Evid Based Complement Alternat Med.

[25] Li H, Venier P, Prado-Álvarez M, Gestal C, Toubiana M (2010). Expression of *Mytilus* immune genes in response to experimental challenges varied according to the site of collection.. Fish Shellfish Immunol.

[26] Mitta G, Vandenbulcke F, Roch P (2000). Original involvement of antimicrobial peptides in mussel innate immunity.. FEBS Lett.

[27] Gestal C, Pallavicini A, Venier P, Novoa B, Figueras A (2010). MgC1q, a novel C1q-domain-containing protein involved in the immune response of *Mytilus galloprovincialis*.. Dev Comp Immunol.

[28] Kong P, Zhang H, Wang L, Zhou Z, Yang J (2010). AiC1qDC-1, a novel gC1q-domain-containing protein from bay scallop *Argopecten irradians* with fungi agglutinating activity.. Dev Comp Immunol.

[29] Zhang H, Song L, Li C, Zhao J, Wang H (2008). A novel C1q-domain-containing protein from Zhikong scallop *Chlamys farreri* with lipopolysaccharide binding activity.. Fish Shellfish Immunol.

[30] Chu FL, La Peyre JF (1989). Effect of environmental factors and parasitism on hemolymph lysozyme and protein of American oysters (*Crassostrea virginica*).. J Invertebr Pathol.

[31] McDade JE, Tripp MR (1967). Lysozyme in the hemolymph of the oyster, *Crassostrea virginica*.. J Invertebr Pathol.

[32] Jollès P, Jollès J (1984). What's new in lysozyme research?. Mol Cell Biochem.

[33] Hong XT, Xiang LX, Shao JZ (2006). The immunostimulating effect of bacterial genomic DNA on the innate immune responses of bivalve mussel, *Hyriopsis cumingii* Lea.. Fish Shellfish Immunol.

[34] Chen Y, Xiang LX, Shao JZ (2007). Construction of a recombinant plasmid containing multi-copy CpG motifs and its effects on the innate immune responses of aquatic animals.. Fish Shellfish Immunol.

[35] Mogensen TH (2009). Pathogen recognition and inflammatory signaling in innate immune defenses.. Clin Microbiol Rev.

[36] Kumazawa NH, Shimoji Y (1991). Plasma-dependent chemotactic activity of hemocytes derived from a juvenile estuarine gastropod mollusc, *Clithon retropictus*, to *Vibrio parahaemolyticus* and *Escherichia coli* strains.. J Vet Med Sci.

[37] López-Cortés L, Castro D, Navas JI, Borrego JJ (1999). Phagocytic and chemotactic responses of manila and carpet shell clam haemocytes against *Vibrio tapetis*, the causative agent of brown ring disease.. Fish Shellfish Immunol.

[38] Stefano GB, Leung MK, Zhao X, Scharrer B (1989). Evidence for the involvement of opioid neuropeptides in the adherence and migration of immunocompetent invertebrate hemocytes.. Proc Natl Acad Sci USA.

[39] Ottaviani E, Franchini A, Kletsas D (2001). Platelet-derived growth factor and transforming growth factor- beta in invertebrate immune and neuroendocrine interactions: another sign of conservation in evolution.. Comp Biochem Physiol Part C Toxicol Pharmacol.

[40] Murray HM, Gallant JW, Pérez-Casanova JC, Johnson SC, Douglas SE (2003). Ontogeny of lipase expression in winter flounder.. J Fish Biol.

[41] Valerio PF, Kao MH, Fletcher GL (1992). Fish skin: An effective barrier to ice crystal propagation.. J Exp Biol.

[42] Chico V, Ortega-Villaizan M, Falcó A, Tafalla C, Pérez L (2009). The immunogenicity of viral haemorragic septicaemia rhabdovirus (VHSV) DNA vaccines can depend on plasmid regulatory sequences.. Vaccine.

[43] De Kinkelin P, Le Berre M (1977). Isolement d'un rhabdovirus pathogène de la truite fario (*Salmo trutta*, L., 1766).. C R Acad Sci Hebd Seances Acad Sci D.

[44] Coll J, Basurco B (1989). Variabilidad del virus de la septicemia hemorragica viral de la trucha en España.. Med Vet.

[45] Saint-Jean SR, Pérez-Prieto SI (2006). Interferon mediated antiviral activity against salmonid fish viruses in BF-2 and other cell lines.. Vet Immunol Immunopathol.

[46] Lorenzo G, Estepa A, Coll JM (1996). Fast neutralization/immunoperoxidase assay for viral haemorrhagic septicaemia with anti-nucleoprotein monoclonal antibody.. J Virol Methods.

[47] Mas V, Pérez L, Encinar JA, Pastor MT, Rocha A (2002). Salmonid viral haemorrhagic septicaemia virus: fusion-related enhancement of virus infectivity by peptides derived from viral glycoprotein G or a combinatorial library.. J Gen Virol.

[48] Reed LJ, Muench H (1938). A simple method of estimating fifty per cent endpoints.. Am J Epidemiol.

[49] Brocal I, Falcó A, Mas V, Rocha A, Pérez L (2006). Stable expression of bioactive recombinant pleurocidin in a fish cell line.. Appl Microbiol Biotechnol.

[50] Tafalla C, Chico V, Pérez L, Coll JM, Estepa A (2007). *In vitro* and *in vivo* differential expression of rainbow trout (*Oncorhynchus mykiss*) Mx isoforms in response to viral haemorrhagic septicaemia virus (VHSV) G gene, poly I:C and VHSV.. Fish Shellfish Immunol.

[51] Falcó A, Chico V, Marroquí L, Pérez L, Coll JM (2008). Expression and antiviral activity of a [beta]-defensin-like peptide identified in the rainbow trout (*Oncorhynchus mykiss*) EST sequences.. Mol Immunol.

[52] Livak KJ, Schmittgen TD (2001). Analysis of relative gene expression data using real-time quantitative PCR and the 2(-Delta Delta C(T)) Method.. Methods.

[53] Pfaffl MW (2001). A new mathematical model for relative quantification in real-time RT-PCR.. Nucleic Acids Res.

[54] Chico V, Gómez N, Estepa A, Pérez L (2006). Rapid detection and quantitation of viral hemorrhagic septicemia virus in experimentally challenged rainbow trout by real-time RT-PCR.. J Virol Methods.

[55] Marroquí L, Estepa A, Perez L (2008). Inhibitory effect of mycophenolic acid on the replication of infectious pancreatic necrosis virus and viral hemorrhagic septicemia virus.. Antiviral Res.

[56] Raida MK, Buchmann K (2008). Bath vaccination of rainbow trout (*Oncorhynchus mykiss* Walbaum) against *Yersinia ruckeri*: Effects of temperature on protection and gene expression.. Vaccine.

