# Supplementary figures and images for: Mytilus galloprovincialis Myticin C: A Chemotactic Molecule with Antiviral Activity and Immunoregulatory Properties
Source: PLoS One. 2011 Aug 8;6(8):e23140. doi: 10.1371/journal.pone.0023140 (PMC3152575; doi:10.1371/journal.pone.0023140)

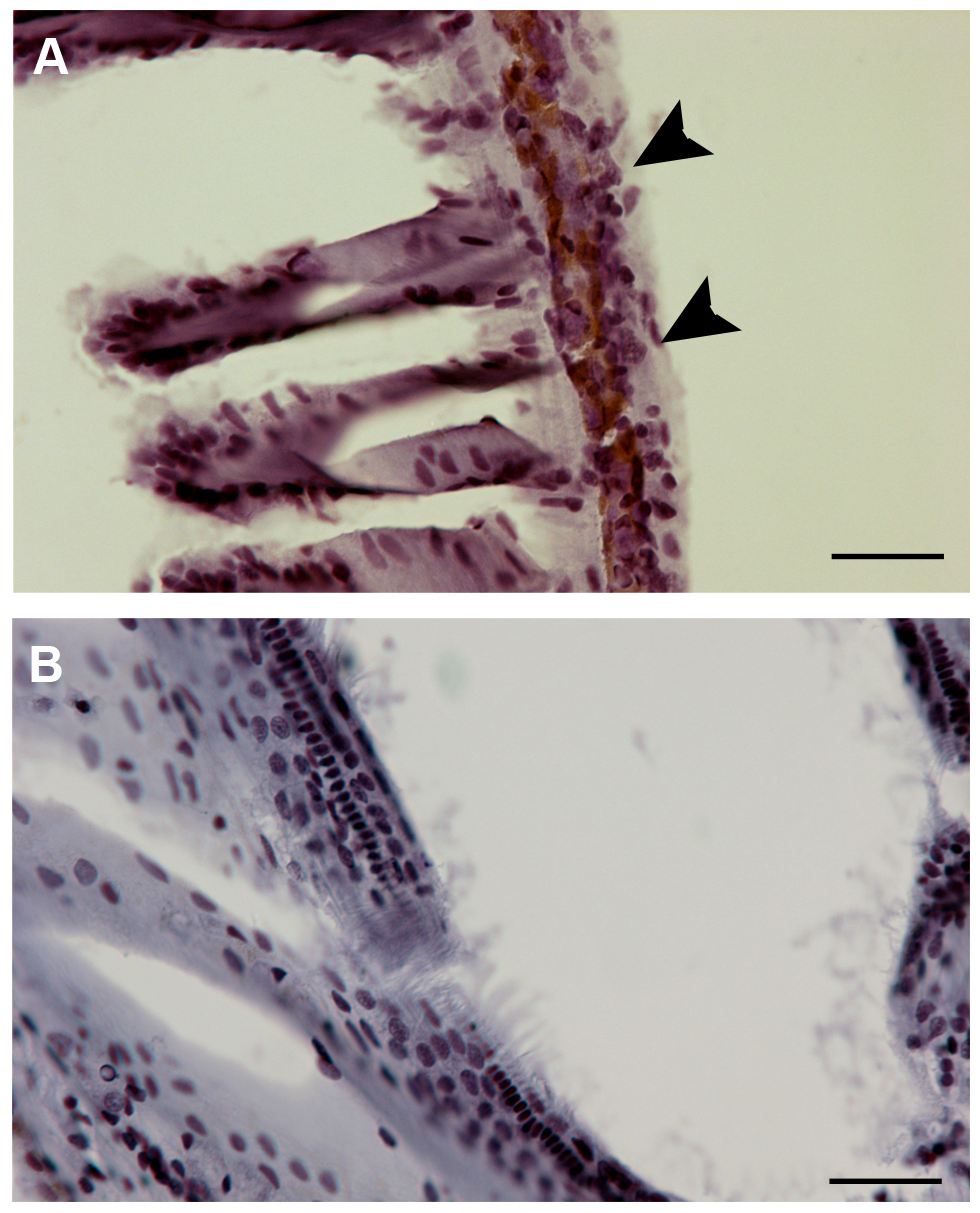

Supplement: Figure S1 — Immunohistochemical determination of the expression pattern of Myt C in gills. Positive hybridization is detected by brown deposits following DAB treatment in A (arrowheads). Control tissues not hybridized with anti-Myticin sera are presented in figure B. Scale bars: 25 µm. (TIF) [file pone.0023140.s001.tif]

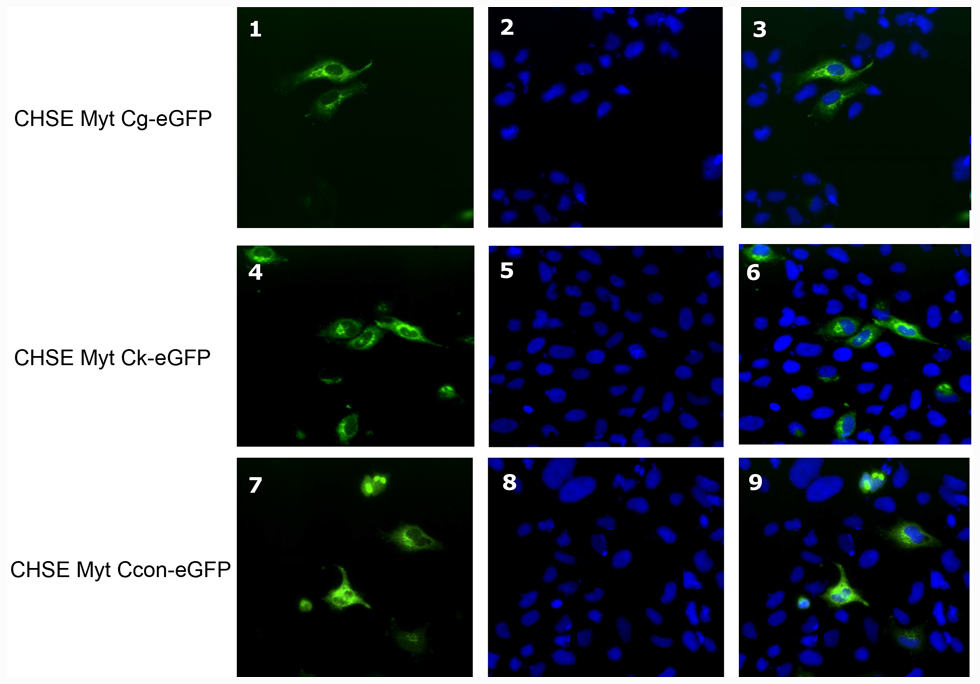

Supplement: Figure S2 — Recombinant expression of Myt C-eGFP variants in CHSE cells as eGFP fusion proteins. CHSE cells were transfected with pMCV1.4-Myt Cg-eGFP, pMCV1.4-Myt Ck-eGFP or pMCV1.4-Myt Ccon-eGFP plasmids and assessed 24 h later. CHSE micrographs with fluorescent (1, 4 and 7) and UV light (2, 5 and 8); merged image of fields 1 and 2 (3), 4 and 5 (6) and 7 and 8 (9), respectively. (TIF) [file pone.0023140.s002.tif]

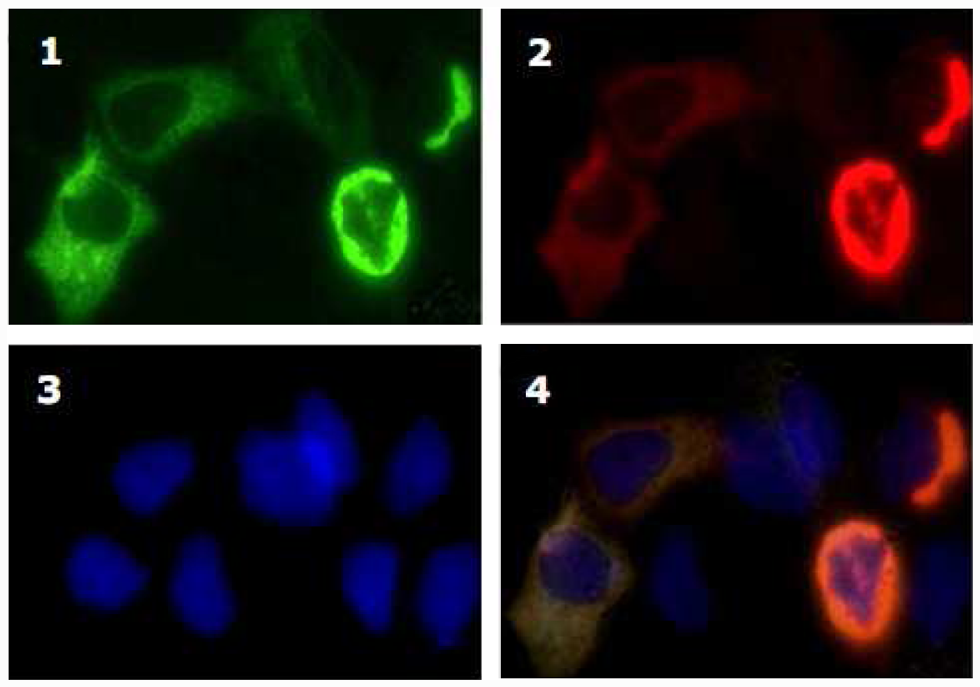

Supplement: Figure S3 — In vitro determination of the subcellular localization of recombinant Myt Cc. Description of data: CHSE cells were transfected with pMCV1.4-Myt Cc-eGFP plasmid and 24 h post transfection washed, fixed and stained with an antiserum anti-Myt C (1), GFP (2) Rho (3) UV (4) merged image of fields 1, 2 and 3. (TIF) [file pone.0023140.s003.tif]
