# Supplementary material for: Mytilus galloprovincialis Myticin C: A Chemotactic Molecule with Antiviral Activity and Immunoregulatory Properties
Source: PLoS One. 2011 Aug 8;6(8):e23140. doi: 10.1371/journal.pone.0023140 (PMC3152575; doi:10.1371/journal.pone.0023140)
Supplement: Table S1 — Nucleotide sequences of Myt C variants and the antisense ISH RNA cDNA template used in this study. (DOCX) [file pone.0023140.s004.docx]

**Table S1. Nucleotide sequences of Myt C variants and the antisense ISH RNA cDNA template used in this study.**

| **Sequence name** | **cDNA Sequence** (5’ to 3’) | **GenBank Accession number** |
| --- | --- | --- |
| Myt Cc | ATGAAGGCAACGATCTTGTTAGCTGTTGTAGTGGCAGTCATTGTTGGAGTTCAGGAAGCCCAATCAGTAGCTTGTAGATCATACTACTGTAGTAAGTTCTGTGGGTCTGCTGGTTGCTCATTATATGGATGTTACCTACTTCATCCTGGAAAAATTTGCTACTGCCTTCATTGTAGCAGAGCTGAGTCTCCATTGGCACTTTCTGGAAGCGCTAGGAATGTGAACGACAAGAACAACGAGATGGACAACTCTCCAGTGATGAATGAGATGGAAAATTTGGACCAAGAAATGGATATGTTCTAG | JF323017 |
| Myt Cg | ATGAAGGCAACGATCTTGTTAGCTGTTGTAGTGGTAGTCATTGTTGGAGTTCAGGAAGCCCAATCAATTCCTTGTACATCATACTACTGTAGTAAGTTCTGTGGGTTAGGTGGTTGCTCATTATATGGATGTTACAAACTTCATCCCGGCAAAATTTGCTACTGCCTTCATTGTCGCAGAGCTGAGTCTCCATTGGCACTTTCTGGAAGCGCTAGGAATGTGAACGAGCAGAACAAAGAGATGGTCAACTCTCCAGTGATGAATGAGATGGAAAATTTGGACCAAGAAATGGATATGTTCTAG | JF323018 |
| Myt Ck | ATGAAGGCAACAATATTATTAGCAGTTCTAGTGGCAGTCTTTGTCGCAGGTATAGGAGCTCATCCGCAAGTTTGCACATCGTACTACTGTGGCAAGTTTTGTGGGACTGCTAGTTGCACACATTATGGATGCCGAAATCTCCATCGCGGGAAACTTTGCTTCTGTGTTCATTGCAGCAGGGTGAAGTTCCCGTTTGGAGCAACTCAAGATGCTAAAAGTATAAACGAACTGGAATACACTCCAATAATGAAGTCGATGGAAAATTTGGACAACGGAATGGATATGTTATAA | JF323019 |
| Myt Ccon | ATGAAGGCAACGATCTTGTTAGCTGTTGTAGTGGCAGTCATTGTTGGAGTTCAGGAAGCCCAATCAGTTCCTTGTACATCATACTACTGTAGTAAGTTCTGTGGGTCTGCTGGTTGCTCATTATATGGATGTTACAAACTTCATCCCGGCAAAATTTGCTACTGCCTTCATTGTCGCAGAGCTGAGTCTCCATTGGCACTTTCTGGAAGCGCTAGGAATGTGAACGAGCAGAACAAAGAGATGGACAACTCTCCAGTGATGAATGAGATGGAACATTTGGACCAAGAAATGGATATGTTCTAG | Consensus |
| As RNA probe | ATATTCCTCAAAACTCAAAACATTCAACATGAAGGCAACGATCGTGTTAGCTGTTGTAGTGGCAGTCATTGTTGGGGTTCAGGAAGCCCAATCAATTCCTTGTACATCATACTACTGTAGTAAGTTCTGTGGGTCTGCTGGTTGCTCATTATATGGATGTTACAAACTTCATCCCGGCAAAATTTGCTACTGCCTTCATTGTCGCAGAGCTGAGTCTCCATTGACACTTTCTGGAAGCGCTAGGAATGTGAACGAGCAGAACAAAGAGATGGACAACTCTCCAGTGATGAATGAGGTGGAAAATTTGGACCAAGAAATGGATATGTTCTAGACAGATATTTGATCAAGAGCTAACTTAGAAAATCAGCTATACTTCTTTCCTTGATGGTGAACAATTTGTGCAAAGTCTGTTGTAAACTTGACAATTTATTAAATCTTCTGTCATACACTTTCGACGTTTTCAGCTTGAA | JF323020 |
